# The optical frequency comb fibre spectrometer

**DOI:** 10.1038/ncomms12995

**Published:** 2016-10-03

**Authors:** Nicola Coluccelli, Marco Cassinerio, Brandon Redding, Hui Cao, Paolo Laporta, Gianluca Galzerano

**Affiliations:** 1 Dipartimento di Fisica-Politecnico di Milano, Piazza Leonardo da Vinci 32, 20133 Milano, Italy; 2 Istituto di Fotonica e Nanotecnologie-CNR, Piazza Leonardo da Vinci 32, 20133 Milano, Italy; 3 Department of Applied Physics, Yale University, New Haven, Connecticut 06520, USA

## Abstract

Optical frequency comb sources provide thousands of precise and accurate optical lines in a single device enabling the broadband and high-speed detection required in many applications. A main challenge is to parallelize the detection over the widest possible band while bringing the resolution to the single comb-line level. Here we propose a solution based on the combination of a frequency comb source and a fibre spectrometer, exploiting all-fibre technology. Our system allows for simultaneous measurement of 500 isolated comb lines over a span of 0.12 THz in a single acquisition; arbitrarily larger span are demonstrated (3,500 comb lines over 0.85 THz) by doing sequential acquisitions. The potential for precision measurements is proved by spectroscopy of acetylene at 1.53 μm. Being based on all-fibre technology, our system is inherently low-cost, lightweight and may lead to the development of a new class of broadband high-resolution spectrometers.

## Abstract (web summary)

An ideal optical frequency-comb system should combine both single-line spectral resolution and a bandwidth broad enough to cover as many lines as possible. Here, the authors incorporate a fibre spectrometer to detect approximately 500 comb-lines with an instrument resolution of 120 megahertz.

## Graphical abstract

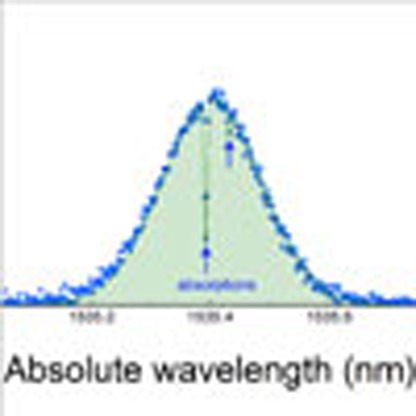

Optical frequency combs (OFCs) are nowadays the preferred tool for frequency measurements owing to their excellent frequency accuracy, high-spectral purity and broad spectral coverage. Originally developed to provide a link between the optical and radio frequency domain^1^,^2^, OFCs have spread in many research areas such as attosecond science^3^, optical waveform generation^4^, remote sensing^5^, microwave synthesis^6^, optical communications^7^ and astrophysics^8^. Presently, OFC systems are well established in the visible and near-infrared (IR) spectral region, and they are expanding into the mid-IR^9^,^10^,^11^, as well as the terahertz^12^, and extreme-ultraviolet^13^ region.

In the frequency domain, the OFC consists of an array of evenly spaced optical lines whose frequencies *v*_*n*_ are defined by *v*_n_=*nf*_r_+*f*_o_, where *n* represents the comb line order, *f*_r_ the comb line spacing and *f*_o_ the comb offset frequency. When both *f*_r_ and *f*_o_ are linked to a frequency standard, the comb is said to be fully referenced and acts as a frequency ruler.

As the OFC has developed over the last years, two general approaches have emerged for its application as an ultraprecise measurement tool. In the first approach, the OFC serves as a frequency ruler against which a continuous-wave (cw) probe laser is referenced^14^,^15^. Once locked to the *n*th comb line with a frequency offset *δf*, the absolute frequency *v*_cw_ of the cw laser can be tracked through the simple relation *v*_cw_=*v*_*n*_+*δf*, and scanned across a molecular absorption by tuning the repetition frequency *f*_r_. This approach is preferred when a single or few absorption lines have to be measured with high precision. The second approach employs the OFC to directly probe atoms and molecules and is called direct comb spectroscopy (DCS)^16^,^17^,^18^. The great advantage of DCS is the large number of comb components providing a massive set of parallel detection channels to gather spectroscopic information. The ultimate requirement on DCS is to push its resolution to simultaneously identify and measure the amplitude of each single mode within the comb. However, this task is highly challenging.

Untill now, scientists have found different techniques for DCS with comb-tooth resolution. A first one is based on the so called virtually imaged phased array (VIPA), that is a side-entrance etalon disperser providing resolution better than 1 GHz (ref. 19). The VIPAs are employed in free-space set-ups that hardly can be integrated in portable devices. This technique allows one to observe up to ∼2,000 separate comb lines (typically 0.2–10 THz bandwidth, depending on comb line spacing) in a single measurement taking a few milliseconds and has been demonstrated in various spectral regions^20^,^21^,^22^. A similar approach is based on substituting the VIPA with a scanning Fabry–Perot (FP) cavity operating according to a Vernier^23^,^24^. This approach has demonstrated near-parallel acquisition of ∼4,000 comb lines (4-THz bandwidth) in a time of 10 ms. A second technique for DCS is called dual-comb spectroscopy, and uses two OFCs with slightly detuned^25^,^26^,^27^ comb-line spacings. This approach has the advantage of useing a single point detector, however, it requires two OFC sources. Dual-comb set-up based on free-running OFCs can only reach resolution of few GHz, that are typically not sufficient for observation of single comb lines^25^. Indeed, pushing the resolution of dual-comb spectroscopy to the comb-tooth level requires complicated set-up to overcome the relative drifts between the two OFCs^28^,^29^; nonetheless, state-of-the-art systems have been shown to resolve ∼120,000 comb lines over 12 THz with acquisition times of 2.7 s (ref. 30). This technique has also been applied to probe two-photon transitions of Rb with Doppler-limited resolution^31^. A third technique, Fourier-Transform Infrared (FTIR) spectroscopy coupled to OFC sources, has become a powerful combination enabling the measurement of broadband spectra with comb-tooth level resolution^32^. As FTIR spectroscopy is based on a Michelson interferometer with a scanning delay line on one arm, reaching comb-tooth resolution requires a sufficiently long delay line (up to 10 m for comb line spacing of 100 MHz), which implies correspondingly long measurement times and extended optical layouts sensitive to vibrations.

In the following, we report on a technique for DCS with resolution at the comb-tooth level. The technique is based on a single OFC source and a fibre spectrometer consisting of a multimode (MM) fibre and a camera for acquisition of images. A fibre spectrometer is fundamentally different from other established spectrometers: while other spectrometers are based on a one-to-one spectral-to-spatial mapping (array spectrometers, VIPAs) or spectral-to-spectral mapping (FTIR, Dual-Comb), a fibre spectrometer is based on a complex spectral-to-spatial mapping where each frequency injected into the spectrometer corresponds to a specific intensity profile (speckle pattern), that is stored in a transmission matrix. A reconstruction algorithm is then used to retrieve an unknown input spectrum based on the measured intensity distribution. The fibre spectrometer was originally proposed as a general purpose device similar to array spectrometers^33^,^34^,^35^ featuring high-resolution operation with spectra constituted by a single line and limited to a span of 12 GHz. The device also allowed for broadband operation, but at reduced resolution of 4 nm. Here, we demonstrate that a fibre spectrometer coupled to an OFC source can be used as a broadband metrology-grade spectrometer for high-resolution DCS experiments. We demonstrate spectral fingerprinting of acetylene at 1.53 μm through parallel acquisition of ∼500 comb lines, corresponding to a span of 0.12 THz, with an instrument resolution of 120 MHz. The span has also been extended to 7 nm (0.9 THz) with unchanged resolution, through sequential acquisition of speckle patterns. We observed absorption lines at signal-to-noise ratio (SNR) of 85 with acquisition times of 10 ms; the overall accuracy on the line centre frequency is ±3.5 MHz. The fibre spectrometer has been tested for amplitude retrieval of comb lines, showing high fidelity and reproducibility at levels of 5%. Moreover, absolute frequency calibration has been demonstrated, preserving the accuracy and precision inherited by the OFC. The resolution of 120 MHz observed with the fibre spectrometer represents a ∼10-fold improvement over state-of-art VIPA-based and free-running dual-comb systems. In addition, as the resolution scales with parameters of the MM fibre such as the length or the numerical aperture (NA), further improvements are possible by tuning the fibre design. Noise sources and instabilities have been reduced, and the overall improved performance avoids time lags due to reconstruction algorithm complexity. The system is simple, robust, long-term stable and can be easily integrated in a compact device.

## Results

### Optical setup, calibration and spectral reconstruction

**Figure 1:**
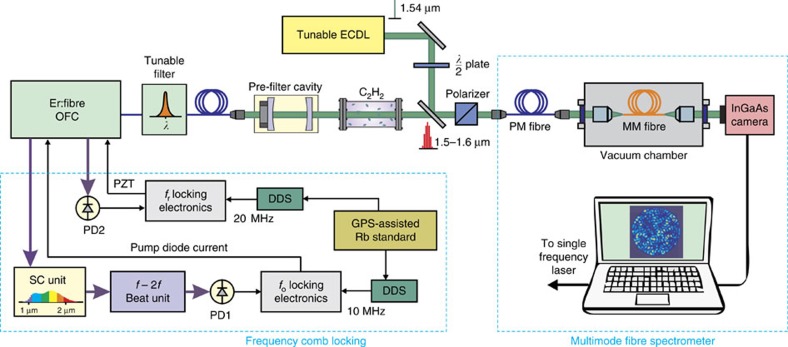
Layout of the experimental set-up for parallel comb spectroscopy. The repetition frequency *f*_r_ and offset frequency *f*_o_ of the Er:fibre OFC are locked to an Rb standard disciplined by a GPS. The OFC output beam passes through the gas cell filled with ^12^C_2_H_2_ at 10 mbar and is multiplexed with the SF laser output beam inside the PM fibre; both beams are coupled into the MM fibre held in the temperature-stabilized aluminium chamber. The speckle pattern emerging from the MM fibre is finally imaged onto the InGaAs camera and processed by a laptop pc. DDS, Direct Digital Synthesizer; ECDL, extended cavity diode laser; PD, photodiode; PZT, piezoelectric transducer; SC, supercontinuum.

The layout of the system used for experiments is shown in Fig. 1. The OFC is based on an Er:fibre femtosecond laser with an output power of ∼0.5 W and a repetition frequency of 250 MHz. Both the repetition frequency *f*_r_ and the offset frequency *f*_o_ of the comb have been locked to an radio frequency reference for long-term stability and absolute calibration of the frequency axis. The bandwidth of the comb is reduced by a fibre-coupled tunable filter having a −10 dB band of ∼0.5 nm. Possibly, the comb lines are also filtered-out with a filtering factor of 4 (or higher) by means of a FP cavity having a free spectral range of 1 GHz (or higher) depending on the comb line spacing required for the experiment. After that, the comb is passed through a 142-mm cell containing acetylene at a pressure of 10 mbar. The comb is multiplexed with a fibre-coupled single-frequency (SF) tunable laser at 1.55 μm that is necessary for the calibration of the spectrometer. The principle of operation of a fibre spectrometer relies on wavelength-dependent speckle patterns formed by interference between the guided modes in a MM optical fibre. In addition to the input wavelength, the speckle pattern also depends on the spatial mode and polarization of light coupled into the MM fibre. To avoid variations in the modes excited in the MM fibre, light is first passed through a polarizing beam-splitter and then coupled to a single-mode, polarization-maintaining (PM) fibre. Finally, the beams are coupled into a standard MM fibre having a core diameter of 105 μm, a NA of 0.22 and a length of 100 m. The MM fibre is housed inside a temperature-controlled aluminium chamber. The speckle pattern at the output of the MM fibre is imaged onto an InGaAs camera with resolution of 320 × 480 pixels.

The output speckle patterns act as a fingerprint, uniquely identifying the spectral components of light coupled into the fibre. To use the MM fibre as a spectrometer, we first calibrated a transmission matrix describing the spectral-to-spatial mapping properties of the fibre (see ‘Methods' section). The transmission matrix *T* relates the discretized input spectrum *S* to a vector describing the discretized speckle pattern *P*, as *P*=*T* × *S* (ref. 35). The calibration is accomplished by tuning the SF laser within the range of interest at proper frequency steps and recording the corresponding speckle patterns produced at the end of the MM fibre, thereby measuring one column of *T* at a time. After calibration, an arbitrary input spectrum can be reconstructed from the speckle pattern it produces as *S*=*T*^−1^ × *P*. Here, the inverse of the transmission matrix was calculated using a singular value decomposition combined with a truncation procedure to discard the weak singular values^35^. Note that the inversion procedure is performed only once as part of the calibration step, after which an arbitrary input spectrum can be reconstructed using a single matrix multiplication, hence the calculation of the spectrum is extremely fast and takes <100 μs on a standard laptop pc.

### Characterization by single-frequency laser illumination

**Figure 2:**
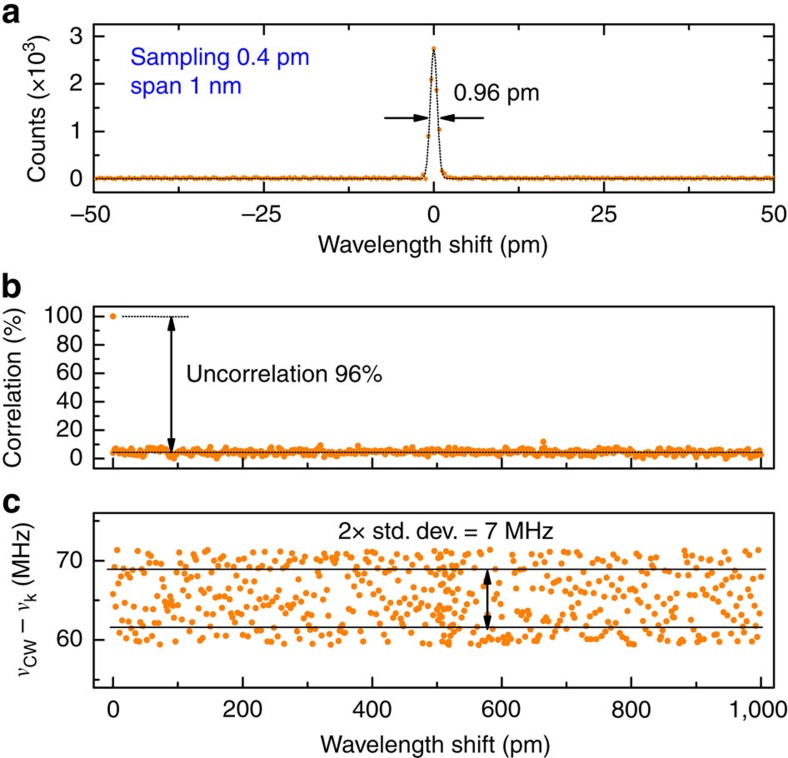
Resolution of the fibre spectrometer and correlation between speckle patterns. (**a**) Spectrum of a narrow-linewidth SF laser at 1,535.3 nm showing the 0.96-pm (120 MHz) resolution of the MM fibre spectrometer. (**b**) Calculated correlation coefficient between the first speckle pattern stored in the calibration matrix, corresponding to 1,535 nm, and all the following patterns acquired during the calibration process by tuning the SF laser at steps of 2 pm (250 MHz) up to 1,536 nm; (**c**) corresponding detuning between the optical frequency *ν*_cw_ of the SF laser and the nearest comb tooth at *ν*_n_, as measured by a frequency counter with gate time of 50 ms.

The fibre spectrometer has been first characterized under SF illumination conditions. The SF laser used for the calibration is used as a probe source of unknown spectrum and its frequency is set at a certain position in the middle of the calibration range. After recording of the speckle pattern, the reconstruction algorithm retrieves the spectrum reported in Fig. 2a, where a band of 0.96 pm can be inferred for the SF laser, limited by the resolution of the fibre spectrometer. Figure 2b shows the correlation coefficient between the first speckle pattern and all the following patterns acquired during calibration at steps of 250 MHz (∼2 pm) in the same spectral range (see ‘Methods' section for details). As clearly shown, all patterns can be considered uncorrelated at a level of 96%; trivially, the uncorrelation level represents the ability of the reconstruction algorithm to separate the different spectral components constituting the spectrum under measurement. Reaching a correlation among all patterns close to zero (ideal condition) requires that the speckles are very sparse and small, and have non-zero intensity at disjunct (not overlapped) pixel positions; depending on the design of the fibre spectrometer, the correlation coefficient can be considerably above zero. It is worth noting that at each step during the calibration, the beatnote frequency between the SF laser and nearest comb tooth is acquired and stored in a separate vector so that it can be used for the absolute calibration of the frequency axis, however, there is no feedback loop to lock the SF laser to the comb. More specifically, the SF laser is scanned through the frequency interval of interest at nominal steps of 2 pm (250 MHz) using a built-in function embedded in the laser software, however, because the SF laser is not locked to the comb, the exact change in frequency at each step is variable. This enormously simplifies the system, as there is no need for feedback loop electronics and controller, hence the calibration process is overall very simple and robust. At the end of the calibration process, there is a complete set of speckle patterns with corresponding absolute frequencies. The vector of beatnote frequencies is shown in Fig. 2c, where it can be seen that all frequencies stay within a range of 14 MHz, with an average value of 66 MHz. This average frequency value is very important for the comb spectroscopy because at the end of the calibration process, the comb is rigidly shifted by the same amount (offset frequency tuning) so that the comb lines stay at the same frequencies where the calibration has been performed.

**Figure 3:**
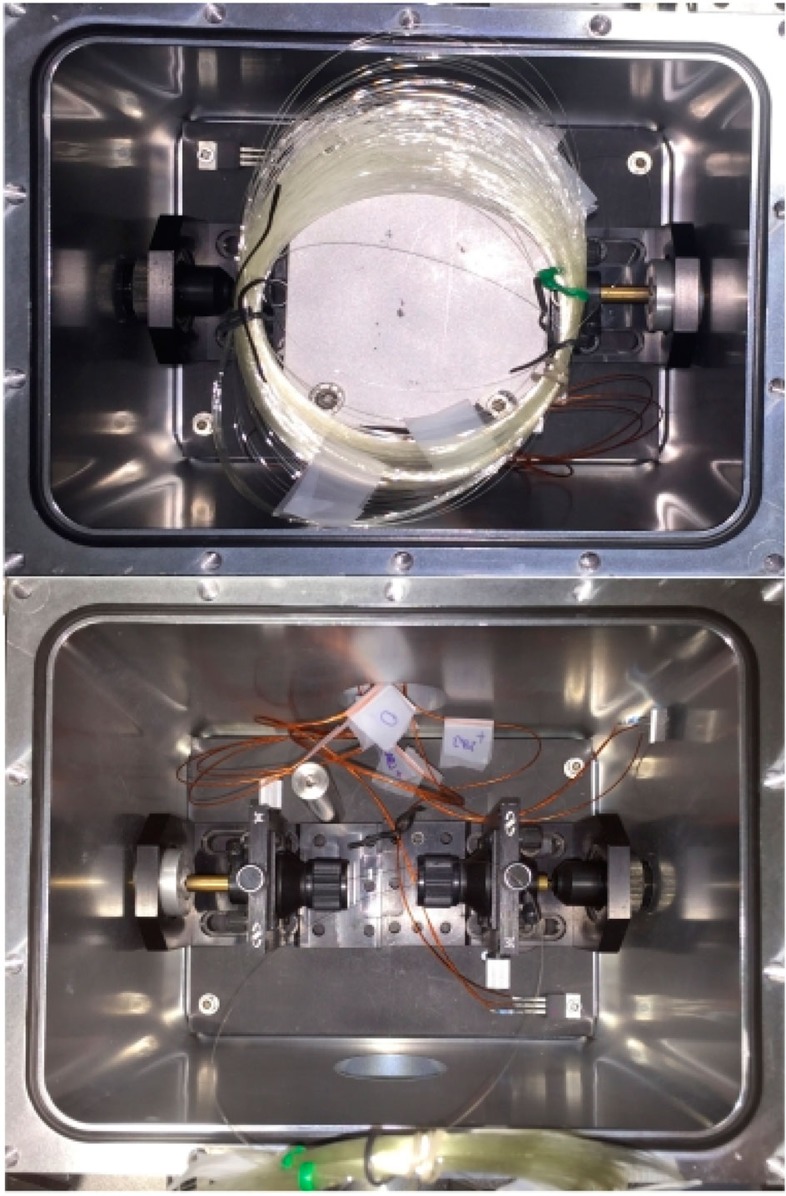
Top-view of the MM fibre and aluminium chamber. The 100-m long MM fibre is coiled and housed in an aluminium chamber (150 × 150 × 220 mm^3^) to provide thermal stability and isolation against external air flowing. Temperature sensors monitor the temperature inside the chamber.

**Figure 4:**
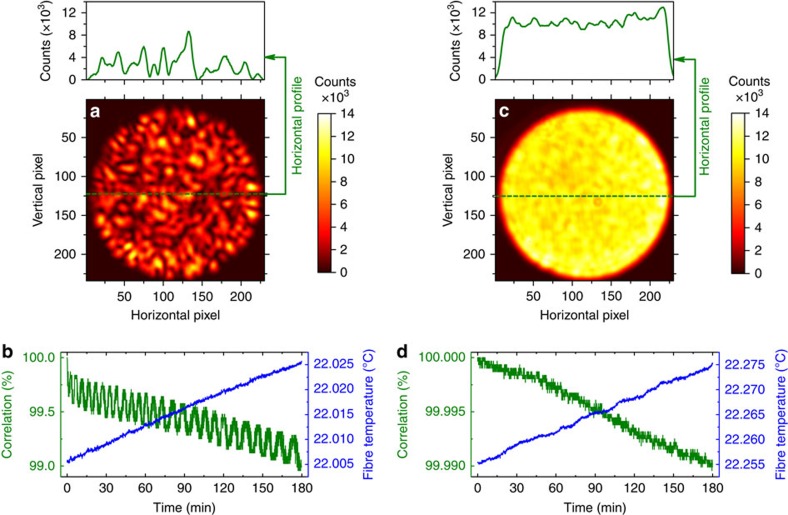
Long-term stability of the OFC fibre spectrometer. (**a**,**c**) Speckle patterns of the narrow-linewidth SF laser and OFC at 1,535.3 nm emerging from the output of the MM fibre and imaged onto the InGaAs camera. Typical values for the speckle contrast (see ‘Methods' section for definitions) of the SF laser and OFC are 0.710 and 0.078, respectively. (**b**,**d**) Autocorrelation coefficient of the speckle patterns and temperature of the MM fibre as a function of time. To avoid frequency drift, the speckle patterns of the SF laser and OFC have been acquired with the laser locked to the OFC which is in turn locked to a Rb standard disciplined by a GPS. A speckle pattern has been stored every Δ*t*=10 s and correlated to the pattern acquired at *t*=0 s; a degradation of nearly 1% and 0.01% after 3 h is observed in the autocorrelation trace of the SF laser and OFC, respectively.

The primary challenge when using such a high-resolution fibre spectrometer is ensuring that the transmission matrix remains stable after calibration. Indeed, the operating principle of fibre spectrometer relies on the same wavelength always producing the same speckle pattern, hence mechanical perturbations or variations in the fibre environment (for example, temperature variations) changing the speckle pattern will corrupt the transmission matrix. In this work, the MM fibre was placed in a temperature controlled, aluminium sealed chamber (see Fig. 3), to minimize environmental perturbations. During experiments, a moderate vacuum condition was kept inside the chamber with a pressure of 10^−3^ mbar; however, it turned out that the key factor to guarantee highly reproducible speckle patterns is keeping the temperature of the chamber (fibre) constant, rather than the vacuum inside the chamber. To characterize the stability of the spectrometer, the SF laser has been locked to the OFC at a wavelength of ∼1,535.3 nm to be sure that its frequency stays constant over a long time, and then a speckle pattern has been recorded every ten seconds and correlated with the first one. The result reported in Fig. 4b shows that the speckle pattern experienced a negligible ∼1% loss of correlation within 3 h, due to the temperature stability of the chamber, which means stable operation for hours after calibrating the transmission matrix. In addition, the excellent fibre stability allowed us to use a simple matrix inversion procedure to reconstruct the input spectrum without the need for the non-linear optimization algorithm used in previous demonstrations of a MM fibre spectrometer^34^,^35^. The ripples in the autocorrelation trace of Fig. 4b could be ascribed to small oscillations of the locking point in the electronics used to lock the SF laser to the comb, but this effect does not influence the spectrum reconstruction process.

### DCS spectroscopy

**Figure 5:**
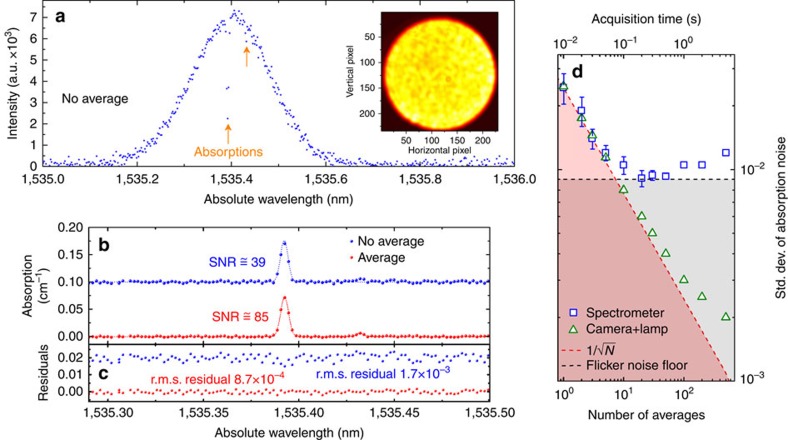
DCS of acetylene and detection limit set by amplitude noise. (**a**) Comb spectrum transmitted by the acetylene gas cell as retrieved by the reconstruction algorithm (no averages) over a span of 1 nm (500 comb teeth) with frequency sampling of 250 MHz. Each circle represents the amplitude of a single comb line. The inset shows the corresponding speckle pattern (contrast of 0.078). (**b**) Absorption coefficient of the P(17) and P(7) lines of the *ν*_1_+*ν*_3_ band of ^12^C_2_H_2_ at 10 mbar. The transmission spectra of the cell have been measured with a single camera exposure (blue circles, offset by 0.1 cm^−1^ for clarity) or by the average of 10 frames (red circles). (**c**) Residuals of the Voigt profile fits to the absorption lines obtained by the single (blue circles, offset by 0.02 cm^−1^) and averaged camera frames (red circles). The main absorption peak observed in the measurement with SNR of 85 (red line) has a line centre wavelength of 1,535.392630±0.000027, nm retrieved from the fit. A secondary absorption peak of amplitude 6.4 × 10^−3^ cm^−1^ is clearly distinguished over the noise. (**d**) s.d. of the absorption noise *σ*_*n*_ as a function of the number of averaged speckle patterns, as measured with the fibre spectrometer operating in standard conditions (blue squares) or by illuminating the camera with a tungsten lamp (green triangles). Also shown the limits set by the camera white noise (red dotted line) and comb flicker noise (black dotted line). The error bars represent the s.d. of *σ*_*n*_ over separate sets of images.

The principle of DCS is based on simultaneously exploiting and demultiplexing the vast number of spectral channels enabled by the OFC. In the ideal case, the system should provide a resolution down to the single comb line level. In this respect, the OFC fibre spectrometer has been tested under broadband illumination condition. Experimentally, the OFC (stabilized to a global positioning system (GPS)-disciplined Rb standard) is passed through a gas cell filled with acetylene (^12^C_2_H_2_) at 10 mbar. The absorption lines of acetylene are very well known (see HITRAN database^36^) and represent a good target for DCS test. The comb is then launched directly into the fibre spectrometer (FP cavity not used), and the transmission spectrum is reconstructed starting from the speckle pattern as reported in Fig. 5. In particular, Fig. 5a represents the transmission spectrum consisting of 500 isolated comb lines measured in a single camera shot with exposure time of 40 μs, with a comb line spacing of 250 MHz. Two absorption lines of the *v*_1_+*v*_2_ band of acetylene, P(17) and P(7), are resolved. Figure 5b shows the corresponding absorption coefficient calculated by the ratio of the transmission spectra of the cell in filled/empty conditions. The single-shot measurement (blue circles) fitted by a Voigt profile is characterized by a notable accuracy on the line centre frequency and amplitude of 0.6 MHz and 5%, respectively. The SNR of 39 obtained in this case is due the high number of comb lines illuminating the spectrometer which causes a reduced contrast (see ‘Methods' section for details) in the measured speckle pattern, hence the camera noise has higher correlations with the speckle patterns at the various frequency components across the calibration range, and the performance of the reconstruction algorithm are limited. Nonetheless, for applications requiring only a quick detection of a specified absorption line, this limited SNR can be sufficient, especially in view of the very short exposure time of 40 μs which could allow for the detection of a fast transient signal via a real-time ‘absorption line movie'. Furthermore, as shown by the averaged multi-shot measurement (red circles) in Fig. 5b, the impact of the camera noise can be easily reduced and a satisfactory SNR of 85 can be obtained by averaging 10 camera frames, which requires an acquisition time of only 10 ms, limited by the maximum frame rate allowed by the camera, that is similar to the DCS techniques with comb-tooth resolution reported so far.

**Figure 6:**
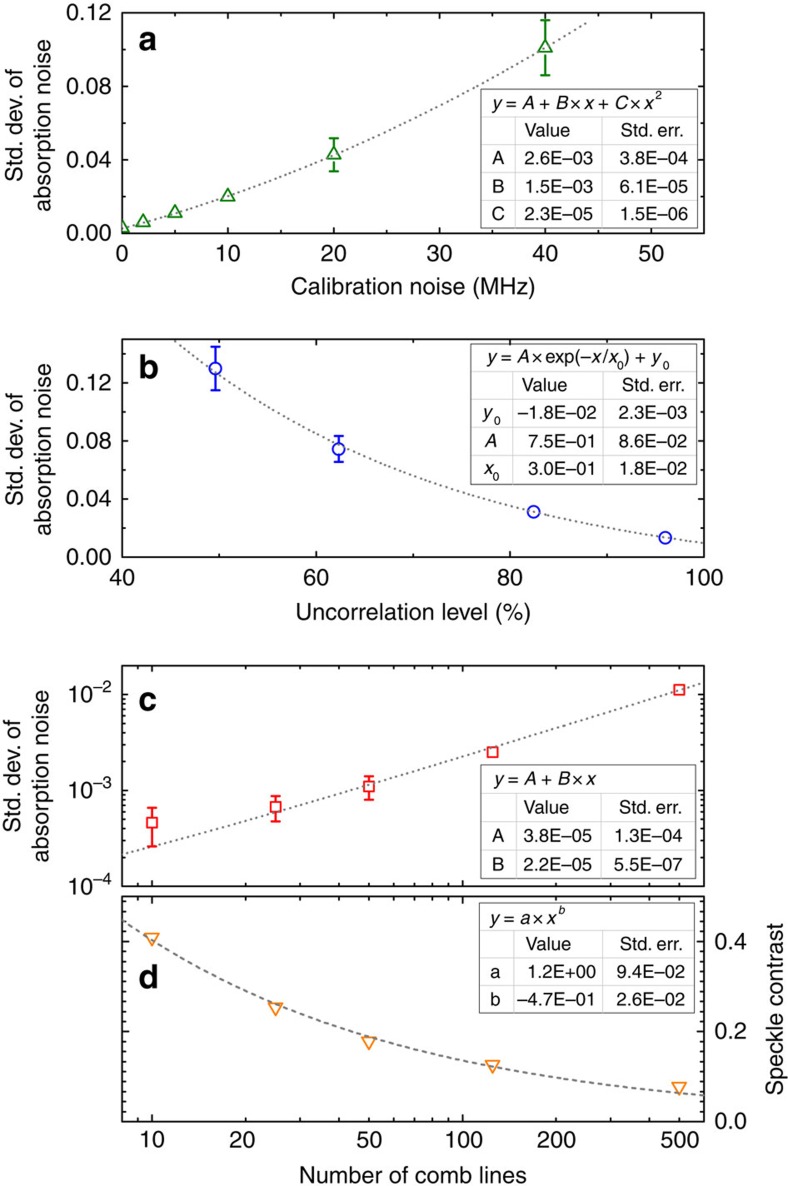
Noise analysis for different noise sources. (**a**) Std. dev. of absorption noise as a function of Std. dev. of calibration noise. The absorption noise has been evaluated by using a high-spectral purity Er:fibre laser locked to the OFC during calibration (see ‘Methods' section for details). (**b**) Std. dev. of absorption noise as a function of the uncorrelation level between the speckle patterns acquired during the calibration of the spectrometer. The uncorrelation has been intentionally degraded by modifying the coupling from the single mode PM fibre to the MM fibre. After calibration, the comb speckle patterns have been acquired and then the absorption noise has been calculated. (**c**) Std. dev. of absorption noise as a function of the number of comb lines in a speckle pattern. The OFC has been filtered by the Fabry–Perot cavity to select a subset of comb lines with desired number of lines. (**d**) Speckle contrast as a function of the number of comb lines. All data have been acquired with speckle pattern averaging set to 10.

There are various noise sources acting on the spectrometer that typically perturb the speckle patterns with the net effect of adding noise to the reconstructed OFC spectra. In particular, a comb speckle pattern can be viewed as the linear combination of speckle patterns corresponding to each comb frequency component. During calibration, the SF frequency laser is scanned through the frequency range of interest to acquire the speckle patterns at frequencies virtually coincident with the comb components (ideal condition) eventually resulting in a spectrum free of reconstruction noise. However, as shown in Fig. 2c, the calibration patterns are acquired at frequencies characterized by a root mean square error of ±7 MHz with respect to the comb components, that is one main source of reconstruction noise, hereafter referred to as ‘calibration noise'. The noise limit set by calibration noise can be quantified numerically in terms of s.d. of absorption noise in the spectra retrieved by the reconstruction algorithm (see ‘Methods' section for details). The results are shown in Fig. 6a. Assuming a near-complete suppression of calibration noise, the absorption noise would be reduced by ∼2.6 10^−3^ with respect to the present condition where calibration noise amounts to ±7 MHz, that is an increase of SNR to ∼384. The calibration noise can be suppressed by scanning continuously the SF laser along the frequency axis (no discrete step), and triggering the camera acquisition when the laser frequency is coincident with a comb component. It should be noted that, similarly to calibration noise, the frequency noise of the OFC, that is, the frequency jitter of each comb component along frequency axis, could be also a possible noise source affecting the reconstructed spectrum. However, the frequency jitter of the comb components is typically much lower than the calibration noise, hence this noise contribution from the OFC is negligible.

As a second source of reconstruction noise, amplitude noise should be considered. More specifically, amplitude noise of the speckle patterns is due to amplitude instability of the comb and electronic noise or drift of the InGaAs camera. Both these noise sources can be attenuated by averaging the speckle patterns. As with calibration noise, the noise limit set by amplitude noise has been quantified by measuring the s.d. of the absorption noise in the reconstructed spectra. The results are shown in Fig. 5d as a function of the number *N* of averaged speckle patterns and the corresponding acquisition time (10 ms per camera frame). The absorption noise decreases as 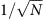 for *N*<20, but is limited at 0.9 10^−3^ (SNR of ∼111) for higher values of *N*. This lower limit is due to flicker amplitude noise in the low-frequency range below 100 Hz of the OFC illuminating the spectrometer; the flicker noise is commonly observed in OFC sources^37^,^38^, and can be even enhanced when the comb offset frequency is stabilized by acting on the pump diode current. However, the flicker noise could be also ascribed to the electronics in the acquisition camera; this has been excluded by performing the measurement of absorption noise with the spectrometer illuminated by a high-stability tungsten lamp (band-pass filtered at 1,530 nm), and observing that the absorption noise follows the 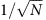 attenuation law even for a number of averages 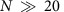, that is where flicker noise of the comb limits the performance.

Among the main noise sources, temperature instability of the MM fibre has to be considered. Changes in temperature affect both the refractive index and the fibre length, and hence the phases of the interfering modes propagating in the MM fibre. As shown in Fig. 4b, a fibre temperature drift of ∼20 mK reduces the correlation of a single speckle pattern by ∼1%. The effect of this can be quantified by looking at Fig. 6b where the absorption noise is plotted as a function of the uncorrelation level between the speckle patterns acquired during calibration. Considering an uncorrelation level of 96%, a reduction by 1% degrades the absorption noise by 1.5̇10^−3^˙1=1.5̇10^−3^, where 1.5̇10^−3^ is the slope of the exponential fitting function around the considered operating point. Hence, a temperature drift of ∼20 mK produces only a minor increase of the absorption noise. On the other hand, assuming a linear dependence of correlation on temperature outside the range shown in Fig. 4b, a temperature drift of 0.2 K would reduce the correlation by 10% and increase the absorption noise by 1.56̇10^−2^. Correspondingly, the SNR in Fig. 5b would be reduced to ∼36, which represents a major increase in reconstruction noise.

Mechanical perturbations of the MM fibre such as acoustic and seismic vibrations, or air flowing, represents another potential noise source. Typically, in laboratory environments, the main concern is related to small fibre deformations or vibrations induced by air flowing, a problem which could be avoided by enclosing the MM fibre in a rigid container such as the aluminium chamber used in this work. For application into environments subject to strong vibrations, the fibres can be fixed with resin so that all the system (fibre+spool+fibre coupling optics) behaves as a rigid body, becoming virtually insensitive to mechanical perturbations^34^,^35^.

The sensitivity of the fibre spectrometer is dependent upon the uncorrelation level of the speckle patterns acquired during calibration. As a test of this characteristic, the absorption noise has been measured at different uncorrelation levels. To this purpose, the uncorrelation has been intentionally degraded with respect to Fig. 2b by acting on the coupling condition of the single-mode PM fibre and the MM fibre. In particular, a small lateral mismatch between the axes of the two fibres excites only low-order modes in the MM fibre, which means larger typical dimensions (radius) of the speckles and hence lower uncorrelation levels; on the other hand, a large lateral mismatch, which is easily tolerated by the MM fibre without affecting the coupling efficiency, allows for excitation of high-order modes yielding higher uncorrelation levels. At each degradation step of the uncorrelation, the calibration procedure has been repeated and then the absorption noise of a typical measurement performed using the OFC has been calculated. The results of this procedure are reported in Fig. 6b. The s.d. of the absorption noise data closely resembles a decreasing exponential function at uncorrelation levels above 40%; for application to DCS experiments, uncorrelation levels above 80% would be preferable.

It is worth noting that the spectral resolution of the fibre spectrometer is virtually not dependent upon the uncorrelation of the speckle patterns, as experimentally verified by reconstructing the spectrum of the SF laser at different uncorrelation levels, and also by observing the correlation traces of the speckle patterns acquired within each calibration procedure performed after changing the uncorrelation level. However, when the spectrometer is illuminated by the OFC and the uncorrelation set to very low levels, the quality of the reconstructed spectra may be sufficiently degraded by the noise that the resolving power of the system would be compromised.

In addition, the dependence of absorption noise has been analyzed as a function of the number of comb lines contained in a single speckle pattern. To this purpose the OFC has been filtered by the FP cavity set to different lengths to select a subset of comb lines with the desired comb line spacing (0.25,1,2.5,5,10,12 GHz) and number of comb lines (*M*=500,125,50,25,10 lines). The absorption noise has been calculated after running the reconstruction algorithm at each different FP cavity lengths, and the corresponding results are shown in Fig. 6c, where a linear dependence on the number of comb lines can be seen. Also, the speckle contrast has been calculated as a function of the number of comb lines; in this case, the data are well fitted by a 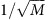 function, *M* being the number of comb lines. Notably, a SNR of ∼1,000 could be reached when the number of comb lines incident onto the spectrometer is limited to 50. Any reduction of the overall absorption noise obtained by acting on the noise sources discussed would allow for increasing proportionally the number of comb lines retrieved in a single acquisition. As an example, a potential ∼4.5-fold improvement of SNR from 84 to 384 could be obtained by suppression of calibration noise from 7 MHz down to a negligible value, as outlined before; at this point, increasing the number of comb lines to 4.5 × 500∼2,250 would restore the SNR to 84 and increase the span to ∼0.5 THz. As another strategy to increase the band, the redundancy of pixels of the acquisition camera could be exploited for parallelization of speckle patterns acquisition without affecting the overall absorption noise (see the ‘Discussion' section for details).

The results presented here have been obtained using a cell characterized by an absorption path length of 142 mm. For extension of the system to situations where very weak absorbers have to be detected or trace-gas measurements performed, the absorption path length can be largely increased by using a multi-pass absorption cell such as a Herriott cell with typical absorption path length in the range 30–200 m, or even a resonant cavity approach, when ultra-high sensitivity is required, reaching equivalent absorption path length up to 10^4^–10^5^ m. As the fibre spectrometer system has been characterized in terms of absorption noise, that is the product of the minimum observable absorption coefficient *α*_min_ and the absorption path length *l*, it is straightforward to calculate the minimum absorption coefficient that could be measured by increasing the absorption path length: as an example, considering the absorption noise of 0.012 obtained in Fig. 5d, a multi-pass cell with absorption path of 200 m would limit the sensitivity of the fibre spectrometer to a minimum absorption coefficient of 0.012/200 m=6 × 10^−5^ cm^−1^. Finally, it should be mentioned that an all-fibre architecture of the system featuring enhanced sensitivity and including also the sample absorption section could be implemented by substituting the free-space absorption cell with a hollow-core fibre enclosed in a vacuum-tight tank filled with the gas sample under analysis; by proper choice of the hollow-core fibre characteristics, the beam generated by the OFC has been demonstrated to propagate with fibre losses of 30 dB ^−1^ km, hence absorption path length of 50–100 m are easily tolerated^27^.

The stability of the spectrometer has been tested using the OFC, and the results are shown in Fig. 4d. In particular, the speckle pattern of the comb in locked conditions has been repeatedly acquired at intervals of 10 s over a time window of 3 h with a resulting overall loss of correlation of 0.01%, that is a factor of ∼100 less than that obtained with the SF laser under similar conditions (that is, similar temperature drift of the aluminium chamber). This result is ascribed to the reduced contrast of 0.078 characterizing the OFC speckle pattern, which is a factor of ∼10 less than the contrast of 0.71 obtained with the SF laser; as the correlation coefficient (see ‘Methods' section for details) depends on the pixel-by-pixel product of two speckle patterns, this leads to a reduction factor of ∼100 in the autocorrelation of the OFC with respect to the SF laser, under similar conditions. The OFC autocorrelation trace does not show the oscillation observed with the SF laser due to the higher long-term stability of the locking electronics dedicated to the comb. Depending on the temperature stability of the laboratory, the calibration might be not preserved over long time window of 24 h.

**Figure 7:**
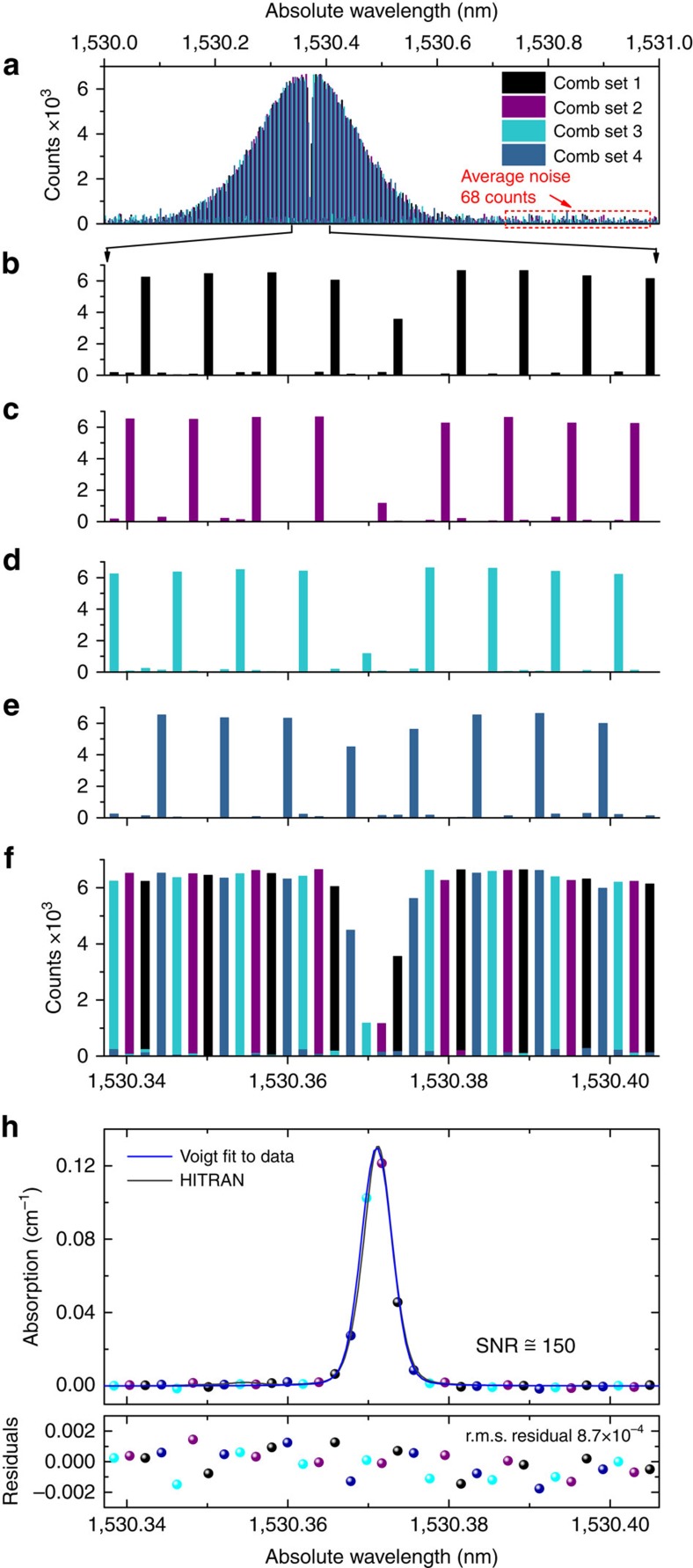
Interleaved DCS demonstrating identification and resolution of each comb line. (**a**) Interleaved comb spectra as retrieved by the reconstruction algorithm over a span of 1 nm (500 comb teeth). (**b**–**e**) Expanded view of the subset of comb lines. The Fabry–Perot cavity transmits one comb tooth over four (attenuation of 25–30 dB out of resonance), resulting in an under-sampled comb with frequency spacing of 1 GHz. The amplitudes and frequencies of the components constituting each sub-comb are accurately retrieved by the reconstruction algorithm. (**f**) Expanded view of the interleaved combs. (**g**) Absorption coefficient as calculated from the ratio between the transmission of the cell filled with ^12^C_2_H_2_ at 10 mbar and in empty condition (blue circles). The absorption data have been fitted by a Voigt profile (blue line) and compared with the absorption data extracted from the HITRAN database. The line centre wavelength with standard error resulting from the fitting is 1,530.371020±0.000021, nm.

The resolution capability of the OFC fibre spectrometer has been investigated in detail. As already shown under SF laser illumination conditions, the fibre spectrometer has a resolution of 120 MHz, that is in principle more than sufficient to resolve single comb lines with our OFC which has a line spacing of 250 MHz. However, under broadband illumination conditions, the contrast of the speckle pattern at the output of the MM fibre is reduced, hence the ability to resolve single lines could be compromised and has to be confirmed. To this purpose, the comb has been filtered by a FP cavity having a free spectral range of 1 GHz to select a subset of lines, and then the transmission spectrum is calculated as reported in Fig. 7b. It can be seen that each comb line is followed by three zero-amplitude lines, that means the comb lines are fully resolved and there is no interference between adjacent components. To further confirm the resolution capability of the spectrometer, the FP resonances have been shifted progressively to select each of the following subset of comb lines and the corresponding spectra have been calculated. As shown in Fig. 7c–e the reconstruction algorithm again provides the expected results. Once the four subsets of comb lines have been acquired, the interleaving of the subsets can be represented as in Fig. 7a,f, where the P(9) absorption line of the *v*_1_+*v*_3_ band of ^12^C_2_H_2_ is clearly visible. Figure 7g shows the Voigt fit to the experimental absorption data and the line profile calculated by HITRAN; the overall agreement in terms of amplitude and frequency is very good. The higher SNR of ∼150 obtained in this case in the Voigt fit is due to the fourfold decrease of the number of comb lines in each speckle pattern leading to sharper patterns characterized by higher contrast.

**Figure 8:**
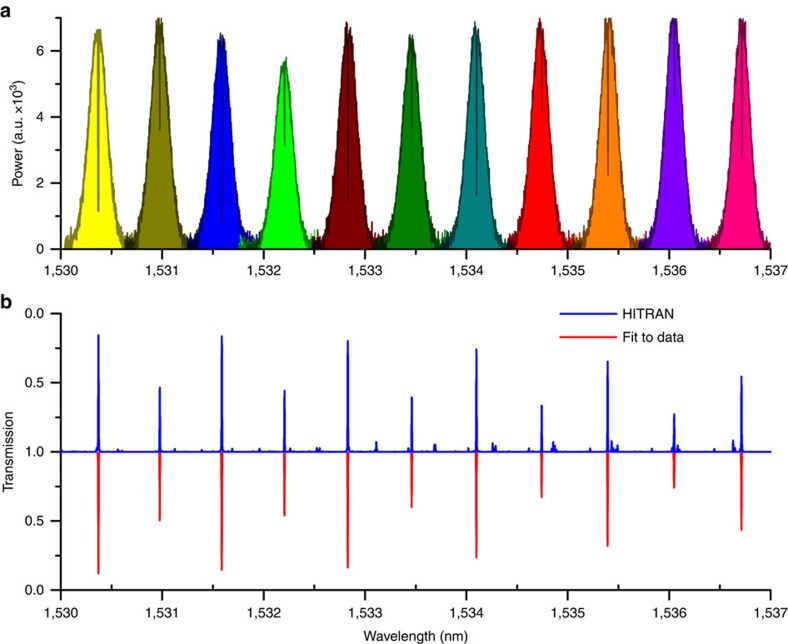
Sequential acquisition of adjacent spectra showing wide spectral coverage. (**a**) A sequence of transmission spectra by a 142-mm cell filled with pure ^12^C_2_H_2_ at pressure of 10 mbar acquired with the MM fibre spectrometer. The sequence has been measured by setting a tunable filter (−10 dB band of 0.6 nm) at the desired central wavelength and imaging the speckle patterns at the MM fibre output onto an InGaAs camera (40-μs exposure time, 10 frames averaged). Each spectrum spans 1 nm and is sampled by ∼500 comb lines. (**b**) Transmission spectrum of the ^12^C_2_H_2_ cell, as calculated by fitting the experimental data corresponding to the main absorptions (red line, bottom) and retrieved from the HITRAN database (blue line, top). The standard error on the centre wavelength of the fitted absorption lines is better than 0.028 pm (±3.5 MHz at 1.53 μm).

The broadband potential of our DCS technique is further demonstrated in Fig. 8, which shows a sequence of spectra corresponding to 11 camera acquisition (averaged over 10 frames) and a total span of ∼0.9 THz (∼7 nm). Each single camera acquisition contains the spectral information from ∼500 comb lines, that means ∼3,500 resolved comb lines over the whole covered span. This acquisition has been possible by calibrating the spectrometer in a single session from 1,530 to 1,537 nm, and then changing the central wavelength of the tunable filter by predefined regular steps, to cover the desired spectral range. At each step of the tunable filter, the camera acquisition is launched and the corresponding spectrum is calculated by a simple and fast matrix product in 10 ms. The red line in Fig. 8b represents the single-line fits to the experimental data, whereas the blue line represents the data from HITRAN. Notably, the line centre frequencies and amplitudes estimated from the experimental data are within 3.5 MHz and 5% (frequency and amplitude accuracy), respectively, compared with data from HITRAN.

## Discussion

While the approach used to extend the bandwidth to 7 nm (as shown in Fig. 8) required a series of sequential measurements, thereby increasing the acquisition time, it is also possible to increase the spectrometer bandwidth in parallel. Specifically, a wavelength-division multiplexer could be used to first divide an unknown input spectrum into a number of subbands^39^. In many spectral regimes (including the telecom band considered in this work) compact, fibre based wavelength-division multiplexer modules are readily available. Each subband can then be coupled to a separate MM fibre spectrometer to record the high-resolution spectrum and the overall spectrum could then be concatenated from the separate measurements. Moreover, by simultaneously imaging the speckle patterns formed on several MM fibres onto a single camera, this method enables one to achieve parallel operation of multiple fibre spectrometers with a single camera. In a fibre spectrometer, the number of pixels on the camera will approximately dictate the number of spectral channels which can be measured (without relying on compressed sensing techniques). Since most 2D cameras have many more pixels (for example, >10^5^) than required for a single fibre spectrometer, which might have ∼1,000 spectral channels, there is significant potential for parallelization.

The resolution of the fibre spectrometer could be further improved to ∼12 MHz. As shown in a previous work^35^, the resolution *δv* of the fibre spectrometer scales as the inverse of the fibre length *L* and the inverse square of the NA (*δv*∝*L*^−1^NA^−2^). Assuming the NA of the MM fibre is changed from 0.22 to 0.5, the resolution would be improved by a factor of 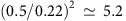. By also increasing the fibre length to 200 m, that is a factor of 2 with respect to the configuration presented in this work, the overall improvement of resolution would be a factor of 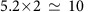. Hence, in the conditions outlined, 500 spectral elements could be simultaneously measured with an instrument resolution of ∼12 MHz, that is nearly two orders of magnitude better then state-of-the-art VIPA systems. However, it is worth noting that, to keep the absorption noise level constant, the span has to be reduced proportionally to resolution, because absorption noise is dependent upon the number of spectral elements resolved.

Finally, although this initial demonstration was performed in the short-wave infrared spectrum, the same principle could be extended to operation in the visible or mid-infrared. The only requirements are to select an OFC and a SF laser source, a MM fibre and an acquisition camera appropriate to the desired spectral region. In particular, OFCs can be generated nowadays in the near- and mid-infrared spectral region with Watt-level powers^10^,^37^,^40^. Quantum cascade lasers represent a reliable and cost-effective technology for the implementation of tunable SF lasers in the mid-infrared up to 18 μm. The technology of infrared fibres based on fluoride (ZrF_4_, InF_3_), chalcogenide (As_2_S_3_, As_2_Se_3_) or polycrystalline (KBl:KCl) materials allows for excellent mechanical flexibility, good environmental stability and high transmission^41^. The best results were achieved with fluoride fibres, which can give propagation losses as low as few dB km^−1^ around 2.5 μm, to be compared with 0.2 dB km^−1^ for silica around 1.5 μm; in the mid-infrared, propagation losses of ∼0.1 dB m^−1^ at 10.6 μm have been demonstrated with polycrystalline fibres^41^. A 100-m long MM fibre could give typical insertion losses of ∼10 dB in the near- and mid-infrared, that is compatible with the power levels of existing OFCs and quantum cascade laser sources. Finally, compact acquisition cameras in the near- and mid-infrared are currently available based on different detector types (InSb, McCdTe) with resolution up to 1,240 × 1,024 and 14-bit amplitude dynamic.

We demonstrated a DCS technique allowing for parallel detection of individual comb lines. The technique is based on a fully referenced OFC source and a fibre spectrometer. We found that a standard MM fibre can be operated as a broadband high-resolution spectrometer by stabilizing its temperature to few tens of millikelvin in a proper container and providing also isolation from air flowing and mechanical perturbations. A 100-m long fibre is sufficient to resolve comb lines with frequency spacing of 250 MHz. This is a simple and viable solution to reach such a high resolution as fibres are cheap, lightweight and can be coiled into small volumes. The performance of the system, tested by precision spectroscopy experiments on acetylene, resulted in estimation uncertainties of the P-branch line centre frequencies at 1.53 μm within ±3.5 MHz and line amplitude within 5%. The absorption lines have been measured at SNR of 85 within a time of 10 ms, with absolute calibration of the frequency axis. Notably, 500 isolated comb lines in a span of 0.12 THz have been characterized with single camera acquisitions, or up to 3,500 comb lines over 0.85 THz, by performing sequential acquisitions at predefined adjacent spectral intervals to cover the band of interest. The span observed with a single camera acquisition can be also scaled to the THz-level by adopting easy fibre solutions. Our system represents arguably the first real application of a speckle-based spectrometer and could inspire a new class of high-resolution, broadband spectrometers and photonic sensors.

## Methods

### Experimental apparatus

A commercial OFC (Menlo Systems, FC1500-250) has been used for the experiments. In particular, the OFC is constituted by a low-power Er:fibre femtosecond oscillator operating at a repetition frequency of 250 MHz, amplified by an Er:fibre amplifier to an average power level of ∼0.5 W in a band from 1,500 to 1,620 nm. A secondary output of the low-power oscillator is also amplified and coupled in a highly non-linear fibre for generation of supercontinuum radiation covering an octave-spanning spectral range from 1,000 to 2,100 nm. The offset and repetition frequency of the comb are locked to a GPS-disciplined Rb frequency standard at 10 MHz (Precision Test Systems, GPS10RBN) by acting on the pump power and piezo cavity mirror of the low-power Er:fibre oscillator. The fibre-coupled tunable filter has a −10 dB bandwidth of 0.6 nm (Santec, OTF-930). The FP cavity (Burleigh, RC-110) is equipped with two mirrors having a radius of curvature of 500 mm, a reflectivity of 99% and a flattened dispersion profile. The FP cavity is locked to the comb by the lock-in technique. The SF laser (Agilent 81600B) used for calibration and testing of the fibre spectrometer is a fibre-coupled semiconductor laser based on an extended cavity design; it can be tuned from 1,440 to 1,650 nm and is characterized by a linewidth of 100 kHz over an observation time of 1 ms. The SF laser was factory-calibrated with accuracy of better then ±*f*_r_/2, *f*_r_ being the repetition frequency of the OFC. Hence, the comb tooth number *n* can be straightforwardly obtained using the relation *n*=*v*_cw_/*f*_r_ where *v*_cw_ is the factory-calibrated frequency of the cw laser. In case the cw laser has to be calibrated, a wave-meter with accuracy better than half the comb line spacing or even few well-known absorption lines of a gas could be used. The MM fibre (Thorlabs, FG105LCA), characterized by a NA of 0.22 and a core diameter of 105 μm, is coiled and housed inside an aluminium chamber with dimensions of 150 × 150 × 220 mm^3^. The temperature of the chamber is stabilized within a range of ∼0.1°C by a standard temperature controller (Lightwave, LDT-5980) and four 10-W Peltier elements. An objective with a NA of 0.5 is used to image the speckle patterns onto a 320 × 256 InGaAs camera (HighFinesse, LC320). During calibration, the SF laser is scanned at steps of 250 MHz (∼2 pm) over a span of 0.12 THz (∼1 nm). Each frequency step takes 300-ms time to let the laser stabilize, and the whole calibration is completed within ∼15 min; the exposure time of the camera is set to 40 μs. All instruments are driven by a laptop PC through a home-made software routine.

### Reconstruction algorithm, correlations and speckle contrast

The MM fibre spectrometer is based on a complex spectral-to-spatial mapping where each frequency injected into the MM fibre produces a unique spatial intensity distribution. This intensity distribution is stored in a transmission matrix which describes the speckle pattern at each wavelength. We calibrated a transmission matrix consisting of 15,625 spatial channels (pixels) and 3,500 spectral channels. The spectral channels ranged from 1,530 to 1,537 nm in steps of 250 MHz (∼2 pm). For each measurement, we used a subset of the measured transmission matrix corresponding to the bandwidth selected by the tunable filter. To reconstruct the original spectra, we adopted a matrix pseudo-inversion algorithm based on singular value decomposition. The singular values below a threshold are truncated to optimize the SNR in the reconstructed spectra.

The speckle contrast *c*_*A*_ of a given speckle pattern *A*_mn_ is calculated as *c*_*A*_=*σ*_A_/*A*, where 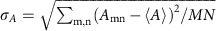 and 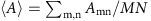, *M* and *N* being the number of rows and columns of *A*_mn_. The correlation coefficient *ρ*_AB_ between two speckle patterns *A*_mn_ and *B*_mn_ is calculated according to the formula 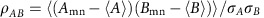.

### Noise limit of the fibre spectrometer

The noise limits of the fibre spectrometer have been characterized in terms of s.d. of absorption noise *σ*_*n*_. Once two spectra *S*_1_, *S*_2_ have been calculated through the reconstruction algorithm in the same conditions, the natural logarithm of the ratio of the two spectra can be expressed as 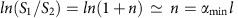 (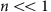), where *α*_min_ represents the minimum measurable absorption coefficient, *l* is the absorption path length and *n* represents the noise of a typical absorption coefficient measurement assuming unitary absorption path length (absorption noise); the corresponding s.d. *σ*_*n*_ sets the fundamental noise limit of the spectrometer. The SNR of a measurement limited by the absorption noise *σ*_*n*_ is given by SNR =1/*σ*_*n*_.

The effect of calibration noise has been measured by using a high-spectral purity Er-fibre laser (NKT Photonics, Basik) during calibration, in substitution of the original semiconductor laser (Agilent 81600B). The Er-fibre laser has a linewidth of ∼3 kHz over 10 ms, and can be only tuned in the range 1,535±0.5 nm. A feedback loop has been added to the system to lock the Er-fibre laser to the OFC and to guarantee that during calibration procedure, the speckle patterns are acquired at exactly the same frequencies of the comb components. This virtually rejects all of the calibration noise and allows for quantifying the noise limit in the optimal condition. Moreover, the calibration noise can be arbitrarily increased by adding a random error (voltage) of proper s.d. in the locking point at each acquisition step of the calibration till reaching the ±7 MHz obtained when using the original semiconductor laser. The absorption noise has been calculated for different s.d.'s of the locking point error in the range from 0 to 40 MHz.

For evaluation of the noise limits set by amplitude noise, the two spectra *S*_1_, *S*_2_ have been calculated from two sequential sets of *N* averaged speckle patterns. The absorption noise has been measured for *N* spanning the range from 1 to 500, corresponding to an acquisition time from 10 ms to 5 s (camera set to 10 ms per frame).

### Data availability

The data that support the findings of this study are available from the corresponding author on request.

## Additional information

**How to cite this article:** Coluccelli, N. *et al*. The optical frequency comb fibre spectrometer. *Nat. Commun.* **7,** 12995 doi: 10.1038/ncomms12995 (2016).
